# Supplementary material for: The interactome of intact mitochondria by cross-linking mass spectrometry provides evidence for coexisting respiratory supercomplexes
Source: Mol Cell Proteomics. 2017 Dec 8;17(2):216–32. doi: 10.1074/mcp.RA117.000470 (PMC5795388; doi:10.1074/mcp.RA117.000470)
Supplement: Supplemental Data [file supp_RA117.000470_133922_0_supp_23978_fzffwf.zip › spectra_annotation/mito_spectra_annotation/190-1-6-1-2-1.pdf]

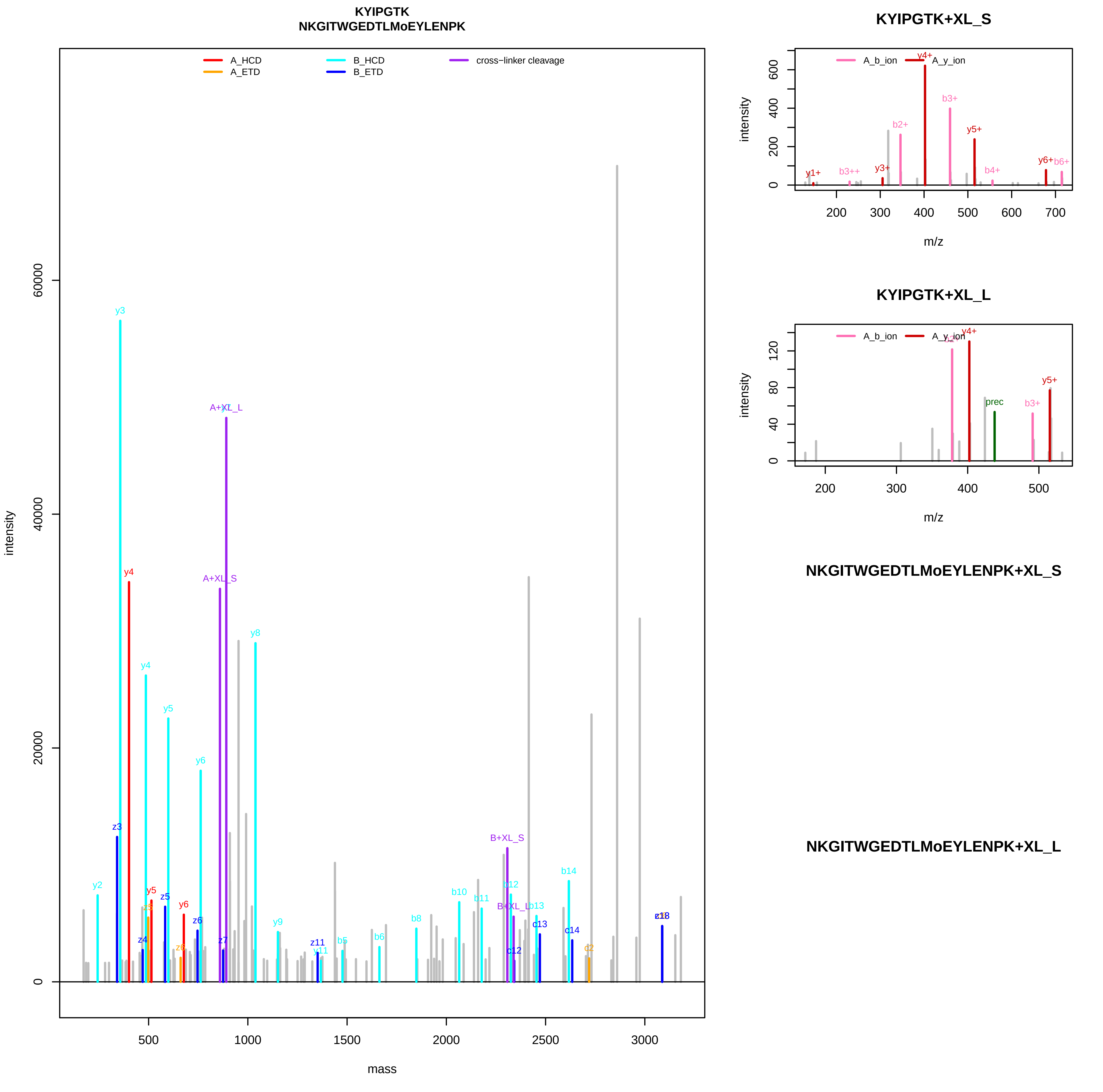
KYIPGTK
NKGITWGEDTLMoEYLENPK
A_HCD
A_ETD
B_HCD
B_ETD
cross−linker cleavage
intensity
mass
A+XL_L
A+XL_S
B+XL_S
B+XL_L
y2 y3 y4 y5 y6 y8 y9 y11
b5 b6 b8 b10 b11 b12 b13 b14
z3 z4 z5 z6 z7 z11
c12 c13 c14 c18
KYIPGTK+XL_S
A_b_ion
A_y_ion
y1+ b3++ y3+ b2+ y4+ b3+ y5+ b4+ y6+ b6+
m/z
KYIPGTK+XL_L
prec
NKGITWGEDTLMoEYLENPK+XL_S
NKGITWGEDTLMoEYLENPK+XL_L
